# Optical Quality and Related Factors in Ocular Hypertension: Preliminary Study

**DOI:** 10.1155/2016/3071036

**Published:** 2016-05-12

**Authors:** Yu-jing Wang, Yan-ning Yang, Lin-ying Huang, Bo Wang, Yu-can Han, Jiang-bo Yan

**Affiliations:** Department of ophthalmology, Renmin Hospital of Wuhan University, Wuhan, Hubei 430060, China

## Abstract

*Background.* To evaluate the optical quality and related factors in patients with ocular hypertension (OHT).* Methods.* This was a prospective case-control study. A total of 12 eyes with OHT and 20 control eyes underwent testing with Optical Quality Analysis System II (OQAS II) to evaluate the modulation transfer function cut off frequency (MTF cutoff), the Strehl 2D ratio (SR), objective scatter index (OSI), tear-film mean OSI (TFOSI), and the OQAS values (OV100%,OV20%, and OV9%).* Results.* The optical quality of patients with OHT declined, with lower MTF cutoff (OHT 36.86 ± 7.11 cpd , controls 48.50 ± 4.04 cpd, *t* = −4.60, *P* < 0.05), lower SR (OHT 0.22 ± 0.04, controls 0.27 ± 0.05, *t* = −2.72, *P* < 0.05), lower OV100% (OHT 1.26 ± 0.25, controls 1.61 ± 0.14, *t* = −4.03, *P* < 0.05), lower OV20% (OHT 1.27 ± 0.27, controls 1.72 ± 0.20, *t* = −4.00, *P* < 0.05), and lower OV9% (OHT 1.30 ± 0.25, controls 1.69 ± 0.32, *t* = −2.28, *P* < 0.05). There were not any statistically significant differences in OSI and TFOSI. The MTF cutoff in patients with OHT was correlated significantly with age (*r* = −0.59, *P* < 0.05).* Conclusions.* Optical quality of patients with OHT is reduced, with lower MTF cutoff, SR, OV100%, OV20%, and OV9%. MTF cutoff is negatively related to age.

## 1. Background

Glaucoma is among the leading causes of blindness in the United States and worldwide which is irreversible [1]. OHT is a leading risk factor for the development of primary open-angle glaucoma (POAG) and the only modifiable risk factor at present [2]. The Ocular Hypertension Treatment Study (OHTS) demonstrated that the cumulative incidence of POAG was 9.5% in the patients with OHT [3]. Medications in controlling IOP may decline the incidence of POAG about 50% but increase the complication of cataract [4].

OHT is the condition with an IOP above 21 mmHg without any treatment or use of medications in the absence of optic nerve damage or visual field loss [5]. The optical quality of patients with OHT is not clearly reported so far. Our study analyzed the optical quality with OQAS II (Visiometrics SL, Spain), which provides parameters such as MTF cutoff, SR, OSI, TFOSI, and OVs. In the meantime, we register the general information of the patients with OHT.

## 2. Methods

### 2.1. Participants

The patients were diagnosed with OHT in Renmin Hospital of Wuhan University from July 2014 to June 2015. In addition, enrolled eyes fulfilled the criteria, including spherical equivalent refractive error from +2.00 D to −2.00 D and cylinder less than 0.25 D; the corrected visual acuity (BCVA) of all subjects was 1.0 or better measured by standard logarithmic visual acuity chart. All people understand and take the initiative to participate in this study. We have the approval of the Ethics Committee of Renmin Hospital of Wuhan University. And the research was in compliance with the Helsinki Declaration.

*Exclusion Criteria*. Exclusion criteria was as follows: (a) dry eyes, keratitis, and other ocular surface diseases; (b) uveitis and vitreous turbidity which influence refractive media; (c) histories of eye surgeries; (d) the use of eye drops within one month. A total of 12 OHT eyes and 20 control eyes were enrolled after signing the informed consent.

### 2.2. OQAS II Measurements

Let the subjects adapt to the dark environment for 3 minutes and then correct the cylindrical defects by external cylindrical lenses; meanwhile the spherical refraction errors were corrected by the double-pass system (±2.00 D). Finally, keep track of the vision quality data for a 4 mm pupil diameter. All subjects underwent three consecutive tests and used the average value. All measurements are performed by an eye specialist.

### 2.3. Ophthalmologic Examinations

Each examination included autorefraction, best corrected visual (BCVA), Schirmer I test (SIt), tear break-up time (BUT) to exclude dry eyes, and B ultrasonic to exclude vitreous turbidity. The intraocular pressure (IOP) and 24-hour IOP was tested by noncontact tonometry (NCT), including the experimental group and control group. Visual field index (VFI) was assessed using a Humphrey Field Analyzer with the central 30-2 program SITA standard. We also observed the Cup/Disc ratio (CDR) with stereoscopic photography and peripapillary retinal nerve fibre layer, central corneal thickness (CCT) by the use of OCT (Cirrus HD-OCT).

### 2.4. Statistical Analysis

Data analysis was performed with SPSS 17.0 (Chicago, IL, USA). After normality testing for continuous variables and Chi-square test for nonparametric comparisons, paired Student's *t*-test was used to compare means of related samples in variables with normal distribution. Paired comparisons included MTF cutoff, SR, OSI, OVs, and TFOSI. Correlation between the MTF cutoff, SR, OSI, and OVs and the age, TFOSI, IOP, CDR, CCT, and VFI was analyzed with Spearman's correlation analysis. The results are expressed as means ± SD, and a *P* value less than 0.05 was considered statistically significant.

## 3. Results and Discussion

A total of 32 eyes were enrolled in the study; 20 were men and 12 were women. The mean age was 25.91 ± 6.31 years (range, 14 to 41 years). All subjects had BCVA 1.0 or better. Most of the clinical characteristics of subjects were summarized in Table 1.

### 3.1. Global Analysis

There were 12 eyes with OHT, 9 males and 3 females, aged 23.33 ± 6.72 years (range: 14 to 32 years old), and 20 control eyes, 11 males and 9 females, aged 27.45 ± 5.67 years (range: 19 to 41 years old). There was no statistically significant difference in gender or age (*P* > 0.05). All the eyes with OHT did not have refractive disorders. The mean spherical equivalent refractive error of manifest refraction in control eyes was −1.32 ± 0.15 D (range, 0 to 1.75 D); the cylinder was −0.07 ± 0.11 D (range, 0 to 0.20 D). It had a statistically significant difference (*P* < 0.05).

### 3.2. Comparability

Table 2 shows the comparative analysis of the variables between eyes with OHT and control eyes about MTF cutoff, SR, OSI, OVs, and TFOSI. It indicated that patients with OHT have significant decline over MTF cutoff, SR, OV100%, OV20%, and OV9%. However, no other variables presented significant differences.

### 3.3. Correlation

We found a significant negative correlation between MTF cutoff and age (Spearman's correlation *r* = −0.59, *P* = 0.04), but there were no statistically significant differences between the MTF cutoff and IOP, OSI, CDR, CCT, and VFI. The data were shown in Table 3.

## 4. Conclusions

### 4.1. IOP Measurement Reliability Analysis

IOP and CCT were found to be positively correlated by several studies [6, 7], but so far, there is no accurate algorithm for correction. Rahman et al. [7] collected measurements of CCT from 1356 normal individuals; the result showed that the CCT is 540 ± 30 *μ*m presenting normal distribution. All subjects in our study were within normal range. There are a variety of tonometers to evaluate IOP, and the GAT is the current reference standard. Cook et al. [8] evaluated differences between NCT and GAT in 15525 participants. The NCT was with the least amount of variability in IOP. Approximately 66% of measurements with the NCT were estimated to be within 2 mmHg of the GAT measurement. Because all the participants in our study need to repeatedly measure intraocular pressure, consideration of cooperation, and simple operation, we chose the NCT.

### 4.2. OQAS

Double-pass technique was put forward by Flamant [9] for the first time in 1955. OQAS based on the technique is the only available device that objectively measures the overall optical quality of human eyes [10] and quantitatively analyze the light scatter and aberration in the optical system. It also provides good repeatability and reproducibility [11]. The OQAS had been extensively used for cataract grading [12] and keratitis, dry eyes [13], laser-assisted in situ Keratomileusis (LASIK) [14], and uveitis [15].

OQAS provides parameters such as MTF, SR, OSI, OVs, and TFOSI to simplify the study of the optical quality of the eye. The MTF represents the loss of contrast produced by the eye's optics as a function of spatial frequency. The MTF cutoff is calculated as that corresponding to a 0.01 modulation transfer function value. It is normally assumed that a cutoff frequency of 30 cycles per degree (cpd) in the Contrast Sensitivity Function corresponds to a visual acuity measurement of 20/20 [16]. The SR is often computed in the frequency domain as the ratio between the volume under the MTF curve of the measured eye and that of the aberration-free eye [17]. The SR of normal people is about 30%. The higher the SR value, the smaller the optical system aberration. The three OVs are normalized values of three spatial frequencies, which correspond to MTF values for three contrast conditions commonly used in ophthalmic practice [18]. The system also quantifies intraocular scattered light by means of the OSI parameter [19]. Values of small OSI are usually linked to eyes with low scattering.

The MTF cutoff and OSI measured in this study were close to others found in similar studies. Our results of MTF cutoff and OSI were 48.50 ± 4.04 cpd and 0.38 ± 0.16 in control eyes, which is consistent with Matinez et al.'s study of 178 healthy eyes range from 18 to 30 years old showed that normal MTF cutoff is 44.57 ± 7.14 cpd and OSI is 0.38 ± 0.19 [20]. Although there were statistically significant differences in spherical equivalent refractive error and cylinder between these two groups, it had little practical influence on the eye's image quality [20].

Compared with control eyes, patients with OHT had lower MTF cutoff, SR, and OVs, which indicated that the contrast sensitivity in OHT is not as good as healthy one. However, the OSI and TFOSI did not show evident distinction, which means the tear-film and the light scatter are not obvious between these two groups. It can be concluded from the results that the difference between OHT patient and normal is mainly reflected in contrast sensitivity.

Contrast sensitivity function has been accepted widely as a sensitive measure for assessing contrast visual performance in various clinical situations [21]. Some reports have demonstrated that contrast sensitivity function is compromised by optics, such as keratorefractive surgery [22]. It is also influenced by retina and brain processing [23]. Contrast sensitivity was significantly reduced in glaucoma patients with newly diagnosed disease and a good visual acuity [24]. Some studies have concentrated on evaluating whether it would be possible to diagnose glaucoma in patients prior to visual field damage using various contrast sensitivity tests [25]. However, researchers have not determined the diagnostic precision of contrast sensitivity to differentiate between OHT and glaucoma. Our study discovered contrast sensitivity changes in OHT by OQAS prior to visual field damage. And these declines may be mainly attributed to optical and retina changes, because OQAS value reflects the light scatter, aberration [11], and retina [26] changes in the optical system. Nevertheless it is difficult to distinguish between true progression to glaucoma and fluctuation unless the test is repeated for a long time. Xu et al. [27] did not find significant change among patients with higher IOP when evaluating optical quality in patients with thyroid-associated ophthalmopathy. We speculated that their result of IOP was influenced by protopathy.

### 4.3. Prospect

There are many common functional and structural investigations in detecting progression from OHT to POAG, such as visual field, stereoscopic photography, and OCT [28]. OHTS found the first evidence of glaucomatous damage through visual field (50%) and the optic disc (40%) among those OHT patients [29], and patients with optic nerve head hemorrhage were more likely to turn into POAG. OCT is a high-resolution cross-sectional imaging technique that allows in vivo measurement of tissue thickness. Research has not determined the diagnose precision of ganglion cell complex (GCC) thickness [30] to differentiate between OHT and glaucoma. In our study, we found a decline in optical quality among OHT, especially the contrast visual acuity; we thought the OQAS could be another measurement for those suspected of having POAG, and it was a more sensitive test to measure the contrast sensitivity than VFI. However, long-term monitoring was needed to find out whether the contrast sensitivity would reduce according to the OHT progress and how many patients with OHT would progress to POAG. In terms of complicated pathogenesis and influential factor of IOP, the sample size in our study is relatively small. A larger number of patients would probably result in stronger significance and sensitivity and specificity values.

In summary, OQAS that has the advantages of easy operation and good repeatability quantitatively analyze the optical quality of OHT. It may be another way to study the pathogenetic mechanism, monitor the progress, and guide medication use in OHT.

## Figures and Tables

**Table 1:** Clinical characteristics of patients.

Case/age/sex	MD	BCVA	IOP	MTF cutoff (cpd)	SR	OV100%	OV20%	OV9%	OSI	TFOSI
Group 1										
1/14/M	OHT	1.0	24.7	51.10	0.28	1.75	1.92	1.84	0.40	0.69
2/14/M	OHT	1.0	24.0	41.44	0.18	1.38	1.34	1.19	0.33	0.56
3/27/M	OHT	1.0	14.3	29.01	0.20	0.97	1.00	1.13	0.37	0.80
4/25/M	OHT	1.0	16.5	31.75	0.20	1.06	1.00	1.10	0.43	0.85
5/28/F	OHT	1.2	16.2	41.36	0.22	1.38	1.43	1.39	0.30	1.45
6/32/M	OHT	1.2	25.3	33.60	0.25	1.12	1.25	1.46	0.34	0.41
7/28/M	OHT	1.5	13.9	40.39	0.28	1.35	1.48	1.63	0.55	0.81
8/28/M	OHT	1.2	23.8	23.52	0.18	0.78	0.88	1.02	0.67	0.88
9/15/M	OHT	1.0	23.1	36.28	0.25	1.21	1.29	1.44	0.34	0.43
10/15/M	OHT	1.0	22.5	40.11	0.21	1.34	1.15	1.18	0.18	0.51
11/27/F	OHT	1.2	23.4	39.26	0.20	1.37	1.21	1.16	0.54	0.90
12/27/F	OHT	1.2	18.6	34.46	0.19	1.36	1.29	1.10	0.41	0.59

Group 2										
1/24/F	Normal	1.0	15.8	47.03	0.23	1.57	1.69	1.26	0.25	0.61
2/24/F	Normal	1.0	10.3	46.23	0.21	1.43	1.57	1.34	0.22	0.45
3/28/M	Refractive error	1.0	19.7	46.27	0.24	1.54	1.57	1.40	0.64	0.89
4/31/M	Normal	1.0	13.2	53.69	0.32	1.79	2.02	2.04	0.28	0.68
5/22/M	Normal	1.0	12.9	50.60	0.24	1.69	1.53	1.34	0.43	1.29
6/22/M	Normal	1.0	11.5	43.59	0.28	1.45	1.40	1.30	0.39	0.74
7/41/F	Refractive error	1.2	18.2	50.81	0.22	1.69	1.78	1.57	0.19	0.02
8/41/F	Refractive error	1.0	11.6	53.15	0.27	1.77	1.95	1.92	0.20	0.28
9/26/M	Refractive error	1.2	13.9	40.57	0.30	1.35	1.54	1.77	0.66	1.13
10/26/M	Refractive error	1.2	17.1	49.90	0.33	1.66	1.79	1.99	0.65	1.09
11/19/M	Refractive error	1.2	12.6	49.34	0.27	1.64	1.72	1.59	0.43	0.34
12/29/F	Refractive error	1.2	12.7	52.67	0.34	1.76	1.95	2.06	0.55	0.36
13/29/F	Refractive error	1.2	14.1	39.85	0.21	1.33	1.32	1.30	0.38	0.59
14/28/F	Refractive error	1.2	16.9	47.95	0.36	1.60	1.86	2.15	0.19	0.81
15/28/F	Refractive error	1.2	12.5	47.54	0.23	1.58	1.68	1.67	0.24	0.42
16/25/M	Refractive error	1.2	14.3	51.69	0.26	1.72	1.83	1.69	0.27	0.44
17/25/M	Refractive error	1.2	14.2	44.63	0.31	1.49	1.64	1.83	0.29	0.44
18/30/M	Refractive error	1.0	11.1	53.16	0.31	1.77	2.06	2.21	0.56	0.69
19/30/M	Refractive error	1.0	11.3	51.25	0.29	1.71	1.87	1.92	0.37	0.43
20/21/F	Normal	1.0	12.7	50.11	0.22	1.67	1.63	1.38	0.34	0.78

Group 1 = patients with OHT; Group 2 = control; MD = main diagnostic; BCVA = best corrected visual acuity; IOP = mean IOP of 24 h IOP; MTF cutoff = modulation transfer function cutoff frequency; SR = Strehl ratio; OV = OQAS values; OSI = objective scatter index; TFOSI = tear-film mean OSI.

**Table 2:** Optical quality of OHT and control.

Project	OHT (12 cases)	Control (20 cases)	*t*	*P*	95% CI	
	Mean ± SD	Mean ± SD			Lower bound	Upper bound
MTF cutoff (cpd)	36.86 ± 7.11	48.50 ± 4.04	−4.60	0.00	−17.44	−6.16
SR	0.22 ± 0.04	0.27 ± 0.05	−2.73	0.02	−0.09	−0.01
OV100%	1.26 ± 0.25	1.61 ± 0.14	−4.03	0.00	−0.55	−0.16
OV20%	1.27 ± 0.27	1.72 ± 0.20	−4.00	0.00	−0.68	−0.20
OV9%	1.30 ± 0.25	1.69 ± 0.32	−2.28	0.04	−0.65	−0.01
OSI	0.40 ± 0.13	0.38 ± 0.16	−0.05	0.96	−0.18	0.17
TFOSI	0.74 ± 0.28	0.62 ± 0.31	0.63	0.54	−0.21	0.37

**Table 3:** Related factors in MTF cutoff.

Related factors	Correlation coefficient	*P*
Age	−0.59	0.04
IOP	0.15	0.64
OSI	−0.39	0.20
CDR	−0.05	0.88
CCT	−0.10	0.76
VFI	0.46	0.13

## Abbreviations

OHT: Ocular hypertension
OQAS II: Optical Quality Analysis System II
MTF cutoff: Modulation transfer function cutoff frequency
OSI: Objective scatter index
TFOSI: Tear-film mean OSI
OVs: OQAS values
SR: Strehl 2D ratio
BCVA: Best corrected visual acuity
IOP: Intraocular pressure
VFI: Visual fields index
OCT: Optical coherence tomography
CCT: Central corneal thickness
CDR: Cup/Disc ratio
POAG: Primary open-angle glaucoma
OHTS: The Ocular Hypertension Treatment Study.
